# Autonomous Driving Control Based on the Technique of Semantic Segmentation

**DOI:** 10.3390/s23020895

**Published:** 2023-01-12

**Authors:** Jichiang Tsai, Che-Cheng Chang, Tzu Li

**Affiliations:** 1 Department of Electrical Engineering & Graduate Institute of Communication Engineering, National Chung Hsing University, Taichung 402, Taiwan; 2 Department of Information Engineering and Computer Science, Feng Chia University, Taichung 407, Taiwan; 3 Department of Electrical Engineering, National Chung Hsing University, Taichung 402, Taiwan

**Keywords:** autonomous driving, deep deterministic policy gradient, recurrent deterministic policy gradient, semantic segmentation

## Abstract

Advanced Driver Assistance Systems (ADAS) are only applied to relatively simple scenarios, such as highways. If there is an emergency while driving, the driver should take control of the car to deal properly with the situation at any time. Obviously, this incurs the uncertainty of safety. Recently, in the literature, several studies have been proposed for the above-mentioned issue via Artificial Intelligence (AI). The achievement is exactly the aim that we look forward to, i.e., the autonomous vehicle. In this paper, we realize the autonomous driving control via Deep Reinforcement Learning (DRL) based on the CARLA (Car Learning to Act) simulator. Specifically, we use the ordinary Red-Green-Blue (RGB) camera and semantic segmentation camera to observe the view in front of the vehicle while driving. Then, the captured information is utilized as the input for different DRL models so as to evaluate the performance, where the DRL models include DDPG (Deep Deterministic Policy Gradient) and RDPG (Recurrent Deterministic Policy Gradient). Moreover, we also design an appropriate reward mechanism for these DRL models to realize efficient autonomous driving control. According to the results, only the RDPG strategies can finish the driving mission with the scenario that does not appear/include in the training scenario, and with the help of the semantic segmentation camera, the RDPG control strategy can further improve its efficiency.

## 1. Introduction

The urban population is growing, and this increase has different outgrowths concerning different topics. For example, the present and further increase in traffic flow is affecting the operational and safety performance of roadways. In accordance with international traffic statistics, the number of daily traveling vehicles amounts to one billion and will reach four billion in 2050 [1]. Hence, automotive safety has become a very critical issue. The automotive safety improvements in the past are all passive safety designs to minimize injuries during accidents, e.g., airbags, shatter-resistant glass, and so on. Then Advanced Driver Assistance Systems (ADAS) actively improve automotive safety by controlling both accelerating/decelerating and steering to reduce the occurrence of accidents and injuries. For instance, in [2,3], the authors propose a car-following model, which works in a human-like way from speed, relative speed, and inter-vehicle spacing. However, ADAS can only be applied to relatively simple scenarios such as highways. If there is an emergency while driving, the driver should take control of the car to deal properly with the situation at any time. Obviously, this incurs the uncertainty of safety. Notice that the main difference between passive and active safety is the operating time [4]. That is to say, an active safety system will operate before the accident and thus attempt to avoid such an accident.

Recently, in the literature, there have been several studies proposed for the above-mentioned issue via Artificial Intelligence (AI). They utilize dynamic simulators with the capacity to accurately and efficiently simulate the behavior of vehicles to conduct experiments and algorithms. The achievement is exactly the aim that we look forward to, i.e., the autonomous vehicle [5,6,7,8]. More specifically, in [5], the authors utilize the transformation in diverse color spaces to design their reward mechanism of Deep Reinforcement Learning (DRL) algorithms. Since the Hue-Saturation-Value (HSV) model is more closely aligned with the color-making attributes of human vision than the Red-Green-Blue (RGB) model, the former is better for color gradations found in nature [9,10]. In [7], the lidar and odometer data are used to obtain the distance and heading information for the designs of their reward mechanism of DRL algorithms. Consequently, we can realize that in the literature, all existing DRL algorithms are only frameworks; it is needed to design and experiment elaborately to implement a viable model based on a certain concept for a specific application.

Likewise, in this paper, we realize autonomous driving control via DRL as well. The CARLA (Car Learning to Act) simulator [11] provides all the necessary components for our experiments, e.g., the car module, the road scenes, the sensed information, and so forth. Specifically, we use an ordinary RGB camera and a semantic segmentation camera to observe the view in front of the vehicle while driving. Notice that since CARLA provides the component of a semantic segmentation camera for the simulation, we do not need to contemplate its implementation; it can be treated as a kind of independent sensor. Then the captured information is utilized as the input for different DRL models so as to evaluate the performance, where the DRL models include DDPG (Deep Deterministic Policy Gradient) and RDPG (Recurrent Deterministic Policy Gradient). Moreover, we also design an appropriate reward mechanism for these DRL models to realize efficient autonomous driving control.

The rest of this paper is structured as follows. At the beginning, the introductory knowledge with regard to this work is reviewed in Section 2, e.g., CARLA, DRL, DDPG, RDPG, and so on. After that, our designs are detailed in Section 3, and the experiments and results corresponding to various experimental settings are performed, analyzed, and discussed in Section 4. Specifically, by utilizing the real-time images of the road in front of the vehicle captured by the ordinary RGB camera and semantic segmentation camera provided by CARLA as the training data, we formulate a proper reward generation architecture with the aim to improve the performance of the adopted models for the autonomous vehicle. Lastly, in Section 5, this work is concluded, and some possible futures are proposed.

## 2. Preliminary

### 2.1. CARLA Simulator

CARLA is an autonomous driving simulator that is open-source and provides the flexible Application Programming Interface (API) to address a wide range of driving tasks. Thus it can help democratize the development of autonomous driving, e.g., driving policies, perception algorithms, and so forth [11].

On the other hand, CARLA is based on Unreal Engine [12] and OpenDRIVE [13] to realize the simulation and define the settings of roads and areas. Noteworthily, Unreal Engine is a research and development tool for real-time technology, which gives creators the freedom and control of virtual worlds. Similarly, OpenDRIVE is used to furnish the road network description, which can be fed into the simulation to develop and validate new techniques.

### 2.2. RGB Camera and Semantic Segmentation Camera

An RGB camera is a camera equipped with a standard sensor through which the color images are acquired. It captures light in red, green, and blue wavelengths for color representation. Since RGB channels roughly follow human vision, it is generally utilized in computer displays.

**Figure 1:**
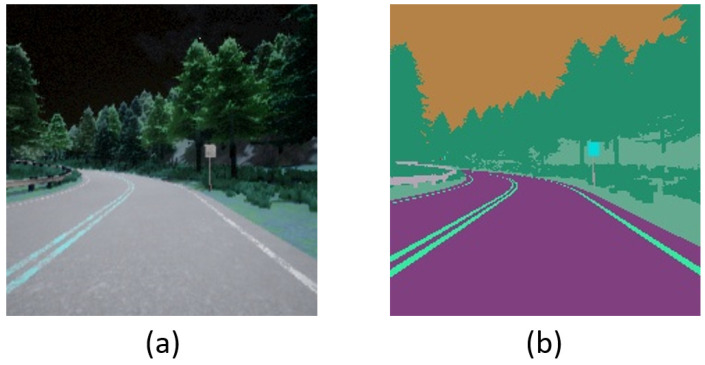
An example of driving vision: (**a**) RGB camera; (**b**) Semantic segmentation camera.

On the other hand, a semantic segmentation camera is very promising and possesses several advantages over conventional cameras [14]. Each object is classified and colored according to its class. For instance, buildings appear in a different color than pedestrians [11]. Moreover, it is a natural motion detector and can filter out redundant information automatically [15]. It can be implemented via various techniques, e.g., Convolutional Neural Network (CNN), Recurrent Neural Network (RNN), Generative Adversarial Network (GAN), and so on. Note that since CARLA provides the component of a semantic segmentation camera for the simulation, we do not need to contemplate its implementation; it can be treated as a kind of independent sensor. Figure 1 shows an example of driving vision obtained by the RGB camera and the semantic segmentation camera.

### 2.3. Constituents of Reinforcement Learning

The main concept of Reinforcement Learning (RL) is the interaction between the agent and the environment. In the process of two-way effect, four primary components need to be considered, i.e., the policy, the reward, the value function, and the environmental model [16,17,18].

- The policy: It is a mapping of all perceived environmental states to actions that can be taken during the procedure. It may involve some extended computations or may be a simple lookup table.
- The reward: After each step, the agent will be rewarded with a single number, which is called the reward. Maximizing the total reward during the procedure is the ultimate object. Noteworthily, a step may simultaneously include more than one action for some particular application.
- The value function: The reward only reveals what decision is better or worse instantly. However, in the long term, the value function can reveal what sequence of decisions is worthier or unworthier. That is to say, for a state, the value is the sum of the reward that an agent may amass from now on.
- The environmental model: The environmental model is utilized to mimic the environmental behavior. Via the environmental model, we can infer the possible future before the experiment is actually performed. Namely, it is utilized for preplanning. Noteworthily, a model-based method needs an environmental model to infer the possible future, while a model-free method is simply trial-and-error.

### 2.4. Model-Based and Model-Free Methods

The model-based method has extreme effectiveness in some applications where the environments are known previously from their rules or designs. Nevertheless, fully model-based cases are rare or may only fit simple and useless scenarios. Namely, the pure tabular solution will limit applicability. On the other hand, the model-free method makes decisions by its knowledge that has been learned via the strategy of trial and error. Apparently, its performance was poor initially due to limited knowledge. However, with continued experiences, it will become more trustworthy. Hence, we can conclude that the model-based method depends on planning while the model-free method depends on learning.

According to the above discussions, we have comprehended that since the environmental model for a model-based solution should be accurate enough to be useful, a model-free solution will have more advantages in the practical and complex applications. That is to say, we will not have the problem of creating a sufficiently accurate environmental model because the model-free solution is used. Noteworthily, the model-free strategy depends on stored values with regard to state-action pairs. They are estimations that the agent can anticipate starting from every action taken at every state. These are acquired via a lot of trials from start to finish. Finally, when the process becomes good enough, the agent will only select the action with the largest action value at every state to make optimal decisions [16].

There are several kinds of model-free branches proposed in the literature, e.g., value-based DQN, policy-based DDPG, policy-based RDPG, and so on. More specifically, since DQN estimates all values for every state-action pair, its performance falls while the considered action space is continuous/complex. Nevertheless, the issue is dealt with easily via DDPG, which possesses the nature of meeting the high dimensional data inherited from Deterministic Policy Gradient (DPG) [19,20]. RDPG is also an extended version of DPG. For solving various physical control problems properly, the memory concept is introduced into DPG. Namely, the short-term integration of information and the long-term integration of memory are both considered in the RDPG approach [21]. In this paper, we will use DDPG and RDPG, combined with different camera data, to implement the autonomous control strategies in Section 3.

### 2.5. Experience Replay

Q-learning, including its successors, can be improved via a method called experience replay [22,23]. It stores the experience at every step in a replay memory. After the emulator executes an action $a_{t}$ in a state $s_{t}$, the reward $r_{t+1}$ and state $s_{t+1}$ will be returned. They form the tuple $\left(s_{t},a_{t},r_{t+1},s_{t+1}\right)$ and are stored in the replay memory. Here, we can accumulate experiences via a lot of plays of the same experiment or game. Then, a mini-batch sampled uniformly and randomly from the replay memory is performed for updates. That is to say, instead of $s_{t+1}$ becoming the update of $s_{t}$, a new unconnected experience is drawn to supply data for the next update.

With the concept of experience replay, Q-learning provides several advantages over its usual form. It allows Q-learning, including its successors, to learn more efficiently. Namely, experience replay reduces the variance of updates and eliminates one source of instability.

### 2.6. Target Network

The concept of the target network is with two opposite networks. One is a target network, which should be relatively stable. The other is being updated continuously. The target network copies the weight from the latter after some predefined steps to avoid the irrelevance of target estimates. Obviously, the former is like offline, and the latter is like online [24].

On the other hand, it can be implemented in another way, i.e., a shared deep learning architecture. Particularly, in the neural network with shared architecture, some common layers are placed on the upper part, and then some split layers are accordingly cascaded onto the lower part for different dedicated purposes. Namely, one is for computing the state value, and the other is for computing the advantage. After that, both are combined to get the action-value approximation.

### 2.7. Policy Gradient

The policy gradient-based approach is very popular, which attempts to leverage the property of differentiability of the policy function for optimization. Particularly, since the policy term is parameterized, it is stochastic. Namely, for a given state, instead of the single best action, the policy may have various probabilities of choosing distinct actions. Hence, it draws stochastic samples to refine the estimate so as to optimize the policy, which may enable the agent to accumulate the maximum cumulative reward [24].

### 2.8. OU Noise

The OU (Ornstein–Uhlenbeck) process is a stochastic process with applications in physical sciences. Originally, it was a model for the velocity of a massive particle under the influence of friction. Noteworthily, the OU process has the property of stationary Gauss–Markov; it may be a Gaussian process, a Markov process, or a homogeneous one [25].

Since OU noise is with the property of time sequence, for the application of vehicle inertia, which is the resistance to change the velocity or direction of a vehicle, either in motion or at rest [26], OU noise is better than Gaussian noise.

### 2.9. DRL Architecture

**Figure 2:**
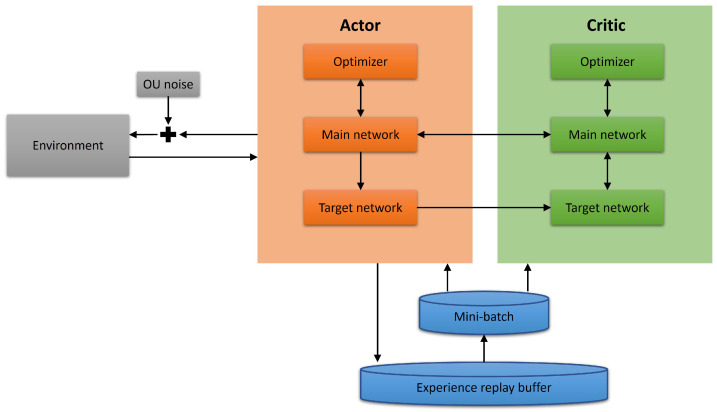
The DRL architecture.

According to the aforementioned descriptions, we can conduct the architecture shown in Figure 2, which includes the experience replay buffer, the introduction of the target network and OU noise, and so on. Owing to the design of the architecture, for various DRL approaches, we only need to replace the source code in the network part with another one.

## 3. The DRL Control Strategies

**Figure 3:**
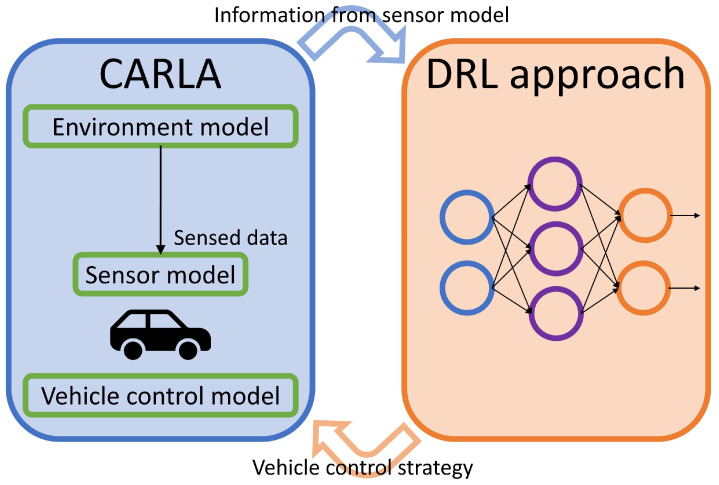
The relationship between CARLA and the control strategy.

Our autonomous driving control strategies are elaborated on in this section. Further, as the description in [5,7], all existing DRL notions proposed in the literature are just frameworks, thus designing a reward mechanism and experimenting with such a mechanism to realize a particular application is still needed. This is the primary contribution of our work. Here, the diagram illustrated in Figure 3 is to demonstrate the relationship between the CARLA simulator and our autonomous driving control strategies. Owing to the design of simulation architecture, for various DRL approaches, we only need to replace the source code in the network part (right component) with another. The CARLA simulator (left component) produces training data that are sent to the DRL approach for the training process. After the training process is finished, the right component is changed to continuously make immediate responses to the left component for controlling the vehicle according to its real-time driving situation.

### 3.1. Designs of Reward Mechanism

In this section, we start to introduce the six primary constituents of the reward mechanism, i.e., $R_{area}$, $R_{roadline}$, $R_{waypoint}$, $R_{velocity}$, $R_{steer}$, and $R_{punishment}$. Noteworthily, the design of the reward mechanism plays an important role in the DRL approach, which affects the performance of convergence as well as the accuracy and stability of the interaction between the agent and the environment.

**Figure 4:**
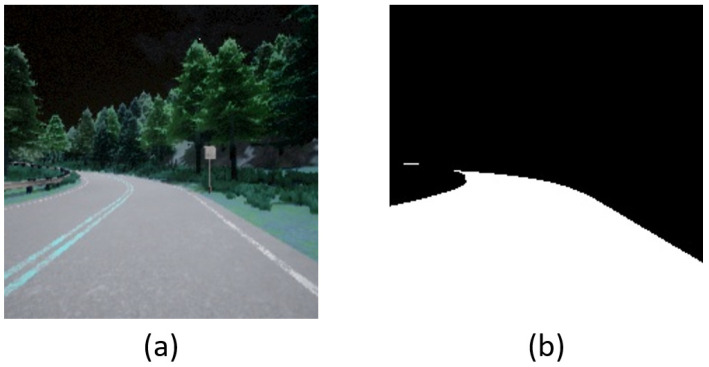
An example of driving vision to obtain $A_{road}$: (**a**) Original; (**b**) Binarization.

First, if a vehicle follows the road stably and accurately, the driving vision will include a higher percentage of the road area. Oppositely, if the driving vision includes a lower percentage of the road area, this means that the vehicle can not follow the road properly. Namely, it may currently be out of control. Hence, according to the notion, the first constituent of the reward mechanism is as follows:

(1) $$R_{area}=\left(A_{road}-T_{road}\right)\times w_{road},$$

where $A_{road}$ is the measure of road area, $T_{road}$ is the threshold, and $w_{road}$ is the weight. Here, $w_{road}$ is used for regulating the ratio of reward. Traditionally, we can use computer vision skill to obtain $A_{road}$. Particularly, since the HSV color model has better efficacy for color gradations found in nature, the first step is to convert the RGB data into HSV color space. After that, binarization replaces each pixel in the driving vision with a white/black pixel [27,28] to obtain $A_{road}$. Noteworthily, thanks to the characteristics of the semantic segmentation camera, in this paper, we can receive $A_{road}$ without the transformation of color space. Figure 4 shows an example of using driving vision to obtain $A_{road}$.

**Figure 5:**
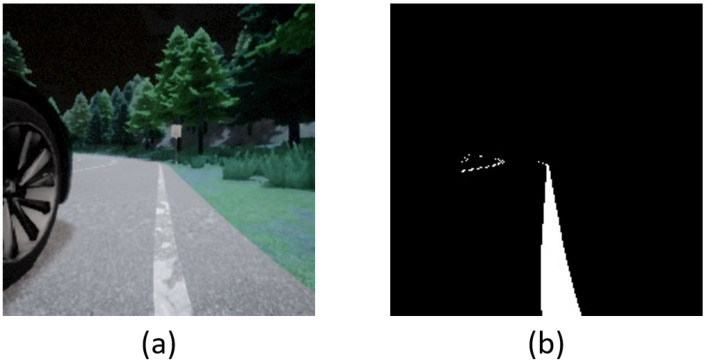
An example of side driving vision to obtain lane lines: (**a**) Original; (**b**) Binarization.

Second, the constituent is $R_{roadline}$, which is used to ensure the keeping of the vehicle inside the lane. If the vehicle stays in its lane, the higher $R_{roadline}$ is rewarded, and vice versa.

(2) $$R_{roadline}=\left\{\begin{array}{c} C_{line}\times w_{line},\left(T_{line,x_{1}}<I_{line,x}<T_{line,x_{2}}\right)\&\left(T_{line,y}<I_{line,y}\right)\\ -C_{line}\times w_{line},otherwise \end{array}\right.,$$

where $C_{line}$ is a constant, $T_{line,x_{1}}$, $T_{line,x_{2}}$, and $T_{line,y}$ are the thresholds of the driving vision, $I_{line,x}$ and $I_{line,y}$ are the current coordinates of the lane lines in the side driving vision, and $w_{line}$ is the weight. Figure 5 shows an example of using side driving vision to obtain lane lines. Notice that the parameters of binarization are different from Figure 4.

**Figure 6:**
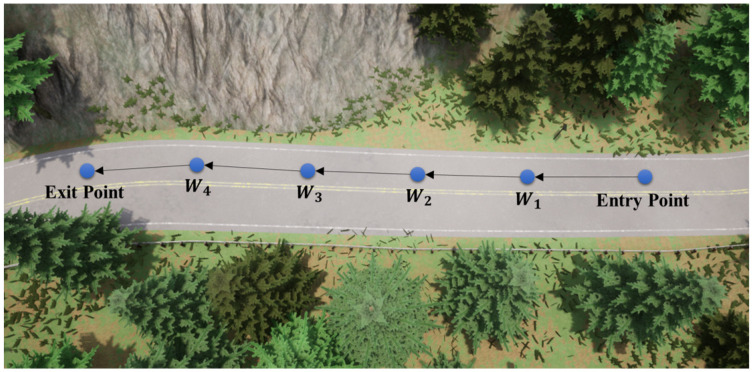
An example of the sequence of waypoints generated via navigation.

The next constituent is $R_{waypoint}$, which is used to ensure correct lane keeping. Because the scenario is right-hand traffic (RHT), the vehicle should keep to the right-hand side lane. Here, the concept of navigation with waypoints is adopted to avoid driving on the wrong side of the road. Particularly, before each trip, the sequence of waypoints will be generated via navigation in advance. Therefore, the vehicle will chase these waypoints for correct lane keeping as well as a higher reward.

(3) $$R_{waypoint}=\left(-\right|P_{vehicle}-P_{waypoint}{|}^{2}+T_{waypoint})\times w_{waypoint},$$

where $P_{vehicle}$ is the position of the vehicle, $P_{waypoint}$ is the position of the next waypoint, $T_{waypoint}$ is the threshold, and $w_{waypoint}$ is the weight. Figure 6 shows an example of the sequence of waypoints generated via navigation.

The next two constituents are regarding the stability of the vehicle, i.e., $R_{velocity}$ and $R_{steer}$. Particularly, if the vehicle speed is slower than a predefined value and the acceleration is not enough, it will be rewarded a negative award. In addition, if the steering angle is similar to that at the latest point in time, it will be rewarded a higher award. Therefore, we can obtain the following two designs for reward constituents.

(4) $$R_{velocity}=\left\{\begin{array}{c} -C_{vel}\times w_{vel},\left(V_{vel}<T_{vel}\right)\&\left(V_{thr}<T_{thr}\right)\\ 0,otherwise \end{array}\right.,$$

(5) $$R_{steer}=\left(\right|S_{t}-S_{t-1}\left|-T_{steer}\right)\times w_{steer},$$

where $C_{vel}$ is a constant, $w_{vel}$ is the weight, $V_{vel}$ is the current velocity of the vehicle, $T_{vel}$ is the predefined threshold of velocity, $V_{thr}$ is the current throttle value of the vehicle, $T_{thr}$ is the predefined threshold of throttle value, $S_{t}$ and $S_{t-1}$ are the steering angles at times *t* and t−1, and $w_{steer}$ is the weight.

The last constituent is the punishment, $R_{punishment}$. Specifically, violating a traffic regulation causes a negative reward and then resets the experiment to the initial status. The constituent is presented below:

(6) $$R_{punishment}=\left\{\begin{array}{c} -C_{punishment},experiment\;reset\\ 0,otherwise \end{array}\right.,$$

where $C_{punishment}$ is a constant.

Eventually, the whole reward mechanism is constructed:

(7) $$R_{total}=R_{area}+R_{roadline}+R_{waypoint}+R_{velocity}+R_{steer}+R_{punishment}.$$

### 3.2. Designs of Actor-Critic Network

**Figure 7:**
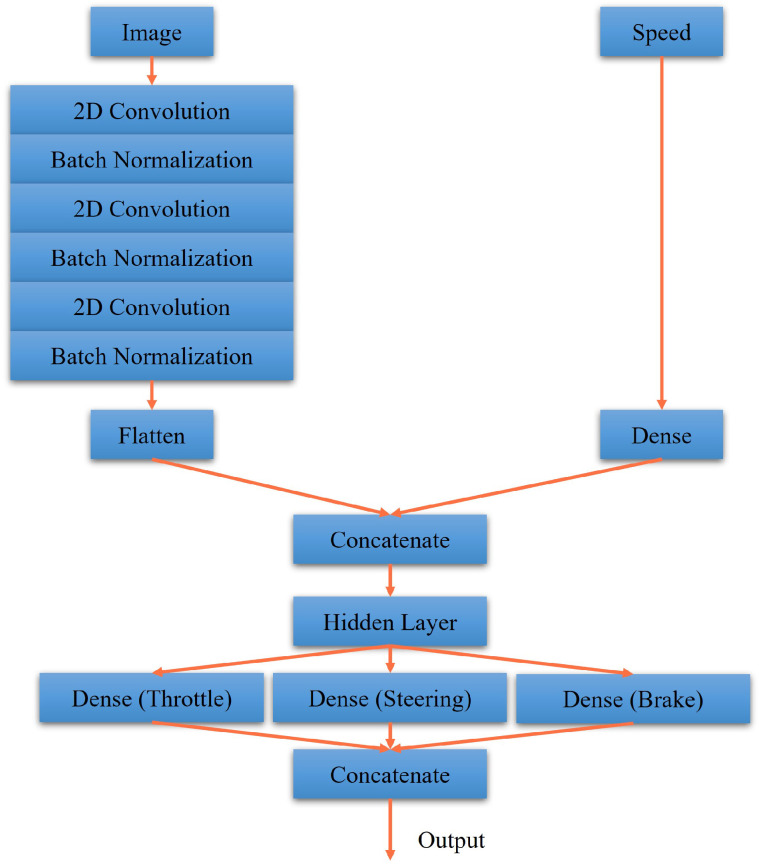
Our actor network for DDPG.

**Figure 8:**
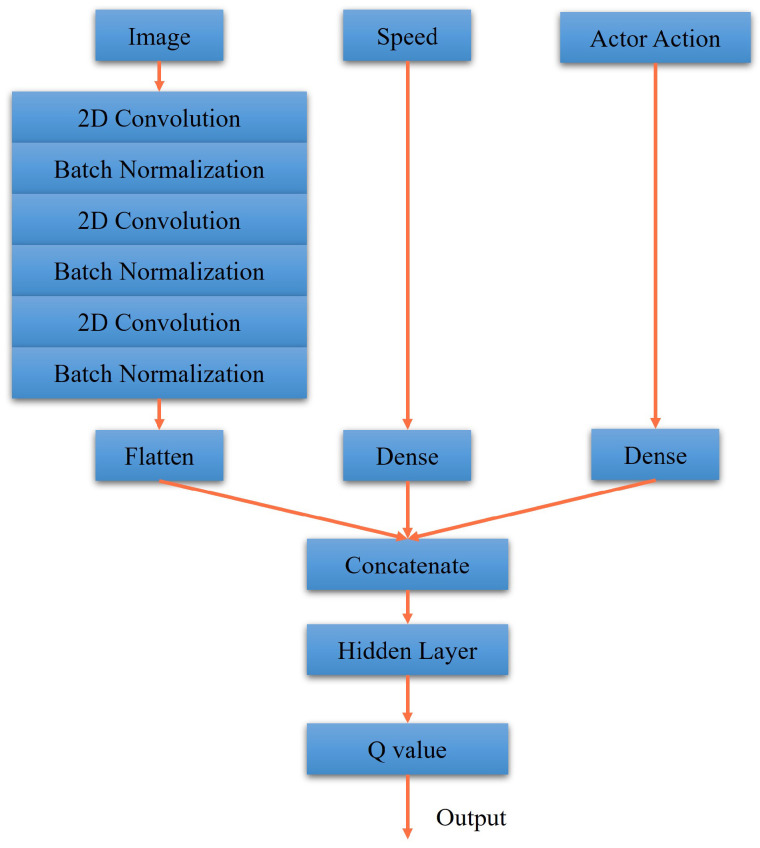
Our critic network for DDPG.

**Figure 9:**
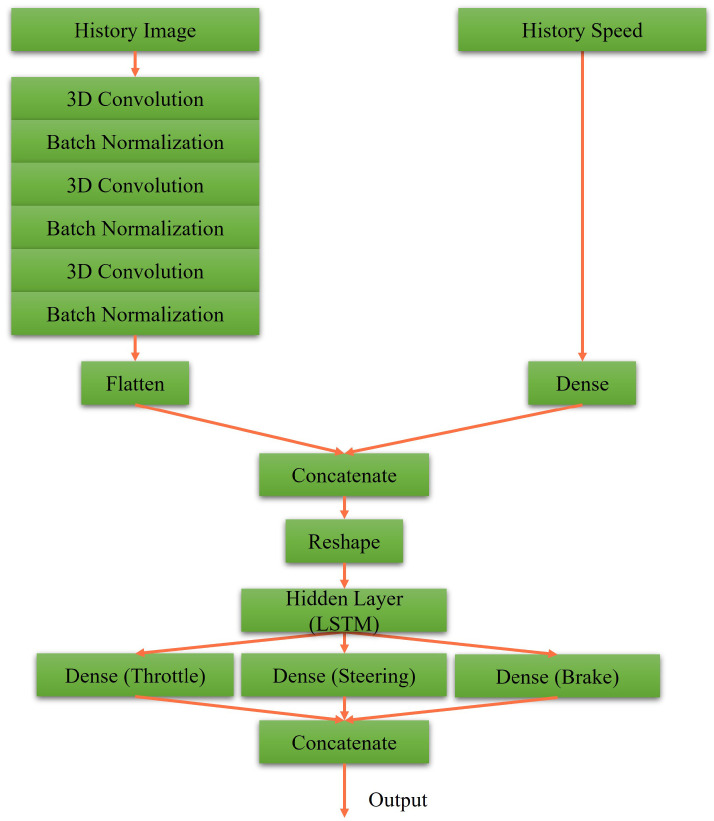
Our actor network for RDPG.

**Figure 10:**
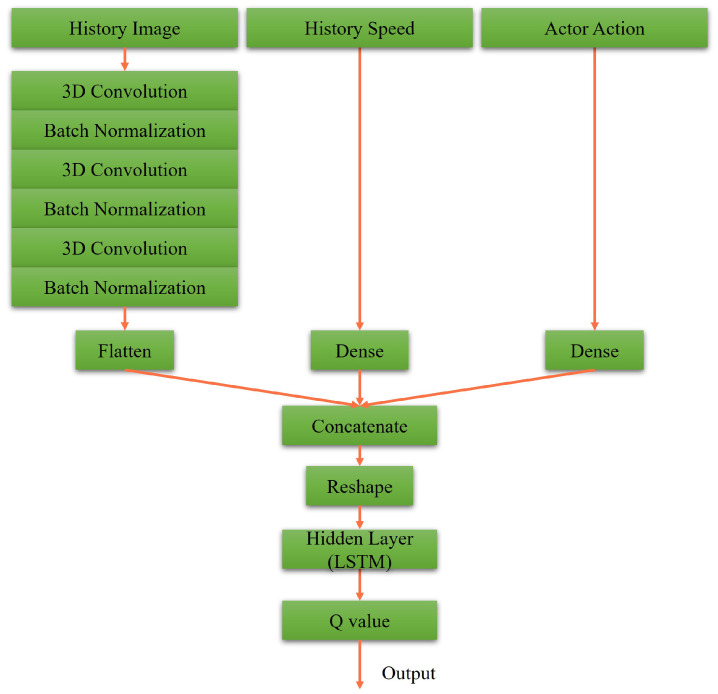
Our critic network for RDPG.

The actor and critic networks of DDPG and RDPG are shown in Figure 7, Figure 8, Figure 9 and Figure 10. First, in Figure 7, the driving vision is taken as the input for the actor network of DDPG. After executing the process of 2D convolution and batch normalization three times, it is concatenated to the driving speed. After that, the concatenated data, including throttle, steering, and brake data, are reweighed as the output. In Figure 8, the critic network of DDPG is presented, where the driving vision, driving speed, and actor action is taken as the inputs. Afterward, they are concatenated and reweighed to obtain the Q value.

Similarly, Figure 9 shows the actor network of RDPG. Particularly, the historical data are taken as the inputs, i.e., driving vision and driving speed. After that, the Long Short-Term Memory (LSTM) layer is utilized to introduce the memory property. In Figure 10, also with the LSTM layer, the historical driving vision, driving speed, and actor action are taken as the inputs. Afterward, they are concatenated and reweighed to get the Q value.

## 4. Experimental Results

**Figure 11:**
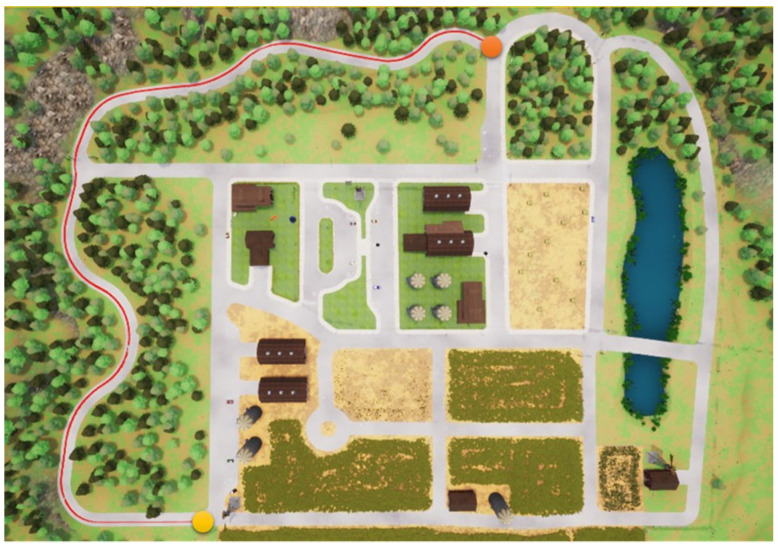
The map used for the training procedures.

In this section, we start to address our experiments in detail. First, the path shown in Figure 11 is used for the training procedure, where the orange point is the starting point and the yellow the destination. In the same map, two other paths are used for the testing procedures. Note that parts of the training and testing paths are mutually exclusive; this is significant and critical for a fair assessment of the experiment. The simulation settings are shown below.

- Two DRL algorithms are adopted for the experiments, i.e., DDPG and RDPG.
- Two kinds of cameras are used to capture the driving vision for the experiments, i.e., RGB and semantic segmentation cameras.
- Hyperparameters:
  - Replay buffer of DDPG: 15,000.
  - Threshold of replay buffer of DDPG: 500.
  - Batch size of DDPG: 150.
  - Replay buffer of RDPG: 6000.
  - Threshold of replay buffer of RDPG: 500.
  - Batch size of RDPG: 150.
  - Learning rate: 0.0001 (actor) and 0.001 (critic).
  - Learning rate decay: 0.9.
  - ϵ from the start: 1.
  - ϵ decay: 0.99.
  - Minimum ϵ: 0.01.
  - τ: 0.005.
  - μ of OU noise: 0.2 (throttle) and 0 (other).
  - θ of OU noise: 0.35.
  - σ of OU noise: 0.1 (throttle) and 0.2 (other).
- The specification of the computer:
  - CPU: Intel Core i7-11700KF.
  - GPU: Nvidia GeForce RTX 3090 24GB.
  - RAM: 48GB DDR4 3600MHz.
  - HDD: 512GB SSD.
  - OS: Windows 10.

Notice that since RDPG has a memory property, less RDPG data occupies the same space as a larger number of DDPG data.

**Figure 12:**
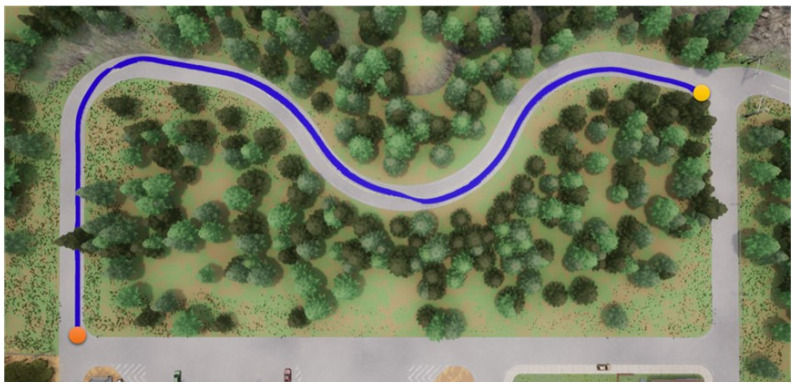
DDPG with an RGB camera for the first testing path.

**Figure 13:**
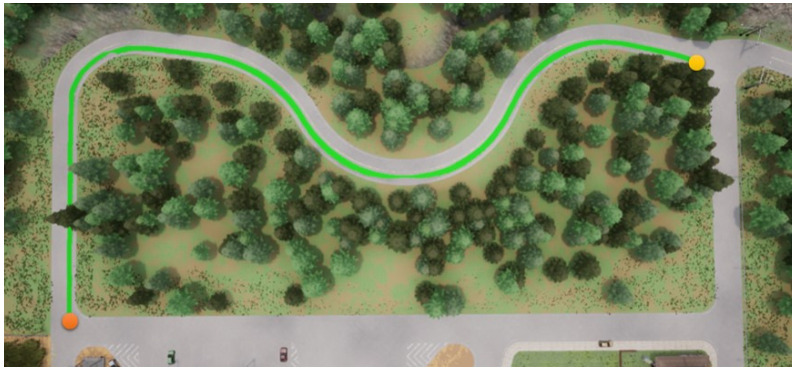
DDPG with a semantic segmentation camera for the first testing path.

**Figure 14:**
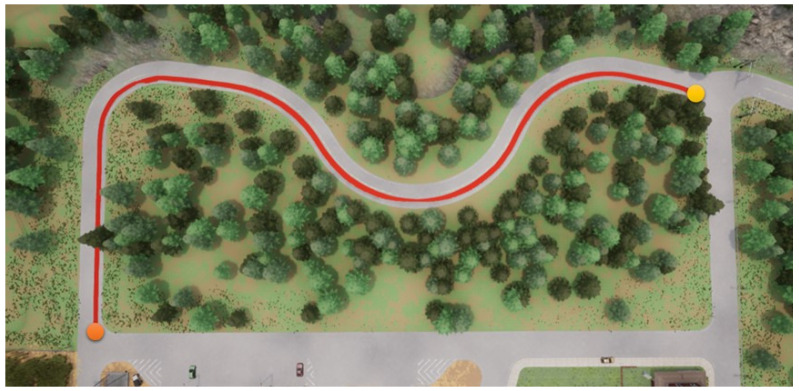
RDPG with an RGB camera for the first testing path.

**Figure 15:**
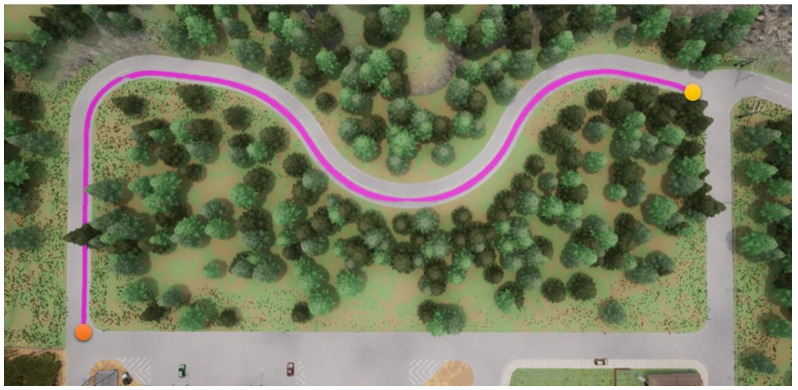
RDPG with a semantic segmentation camera for the first testing path.

**Figure 16:**
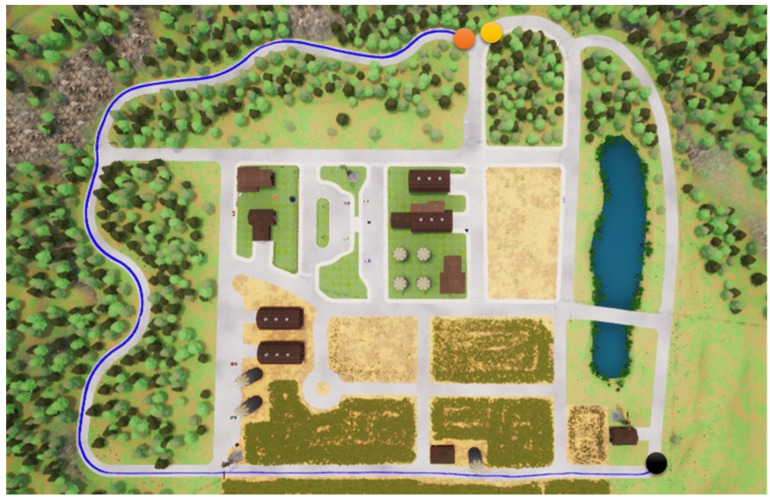
DDPG with an RGB camera for the second testing path.

**Figure 17:**
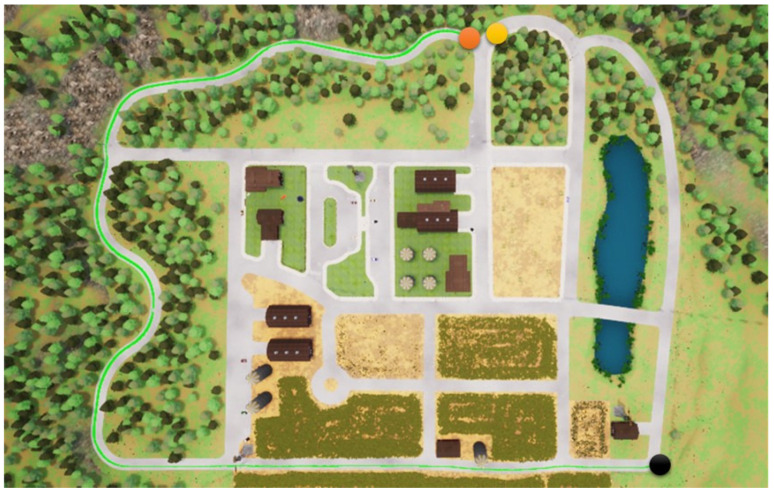
DDPG with a semantic segmentation camera for the second testing path.

**Figure 18:**
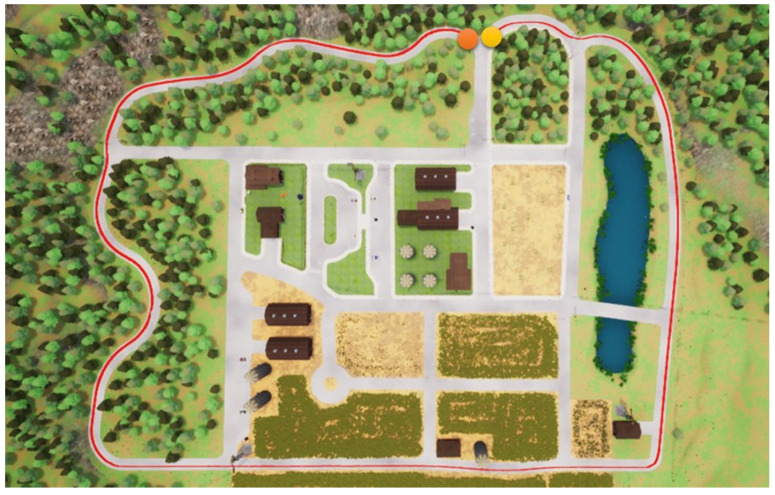
RDPG with an RGB camera for the second testing path.

**Figure 19:**
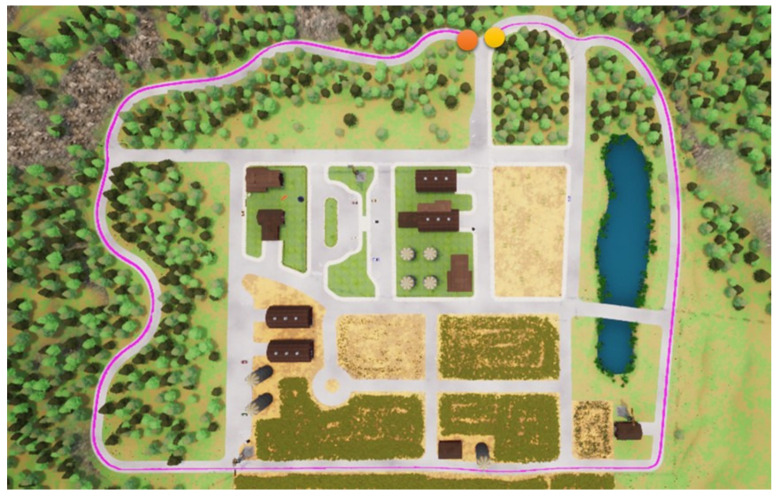
RDPG with a semantic segmentation camera for the second testing path.

Foremost, the first testing path is simpler and shorter, and the corresponding results are presented in Figure 12, Figure 13, Figure 14 and Figure 15, where the orange point is the starting point and the yellow the destination. We can observe that the four autonomous vehicles based on different strategies all finish the driving mission. After that, the second path is more complex and longer, and the corresponding results are presented in Figure 16, Figure 17, Figure 18 and Figure 19, where the orange point is the starting point and the yellow the destination. Here, only two autonomous vehicles based on the RDPG strategies finish the driving mission. Two autonomous vehicles based on the DDPG strategies do not pass the scenario of an L-turn (both fail at the black point), which does not appear/include in the training scenario. According to the aforementioned results, we can conclude that since RDPG has the memory property of adopting inputs in sequence, it has better adaptability for a scenario that the autonomous vehicle has never seen in advance.

**Figure 20:**
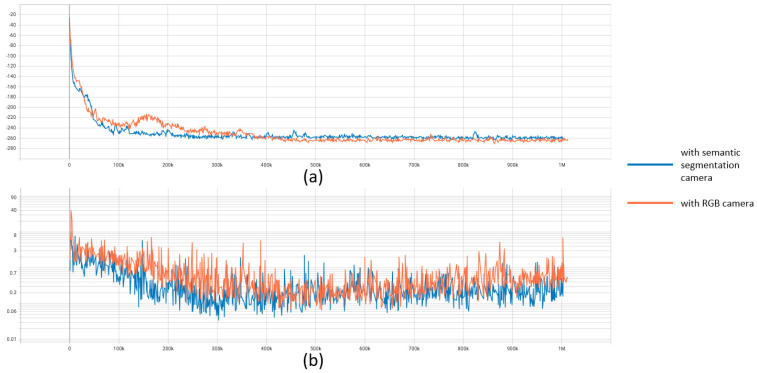
(**a**) The actor loss of RDPG during the training procedure; (**b**) The critic loss of RDPG during the training procedure.

Next, we further discuss the training performance of two RDPG strategies that have better adaptability in the above experiments. The results are presented in Figure 20, where the actor loss and critic loss of RDPG during the training procedure are given. According to the data of the actor loss and critic loss, we can observe that RDPG with a semantic segmentation camera has better convergency performance. Even RDPG with a semantic segmentation camera has a lower loss value at the end of the training procedure. Hence, we can conclude that with the help of a semantic segmentation camera, RDPG can further improve its efficiency.

## 5. Conclusions and Future Work

In this paper, we have used an ordinary RGB camera and a semantic segmentation camera to observe the view in front of the vehicle while driving. The captured information has also been utilized as the input for different DRL models so as to evaluate the performance, where the DRL models include DDPG and RDPG. Moreover, we have designed an appropriate reward mechanism for these DRL models to realize efficient autonomous driving control. According to the results, only the RDPG strategies can finish the driving mission with the scenario that does not appear/include in the training scenario. Next, we have further compared the RDPG strategies with different cameras, and the results have shown that with the help of a semantic segmentation camera, RDPG can further improve its efficiency.

For future work, we plan to propose some new methods with asymmetric architecture for achieving better performance. Namely, some data may be checked continuously, but some may be periodic. According to this asymmetric architecture, we will have less computational complexity and better convergence performance. On the other hand, for different types of vehicles, the consideration of cameras with different angles will be another critical topic for DRL control strategies.

## Data Availability

Not applicable.
